# Characteristics of cold-induced vasodilation among Tibetans and Han Chinese at high altitudes

**DOI:** 10.1186/s40101-025-00404-8

**Published:** 2025-09-30

**Authors:** Hong-Chen Xie, Qi Cui, Shen-Wei Xie, Wen-Jun Jiang, Xiang-Qiong Meng, Ming-Hai Zheng, Xiao-Yong Huang, Xiao-Ling Tan

**Affiliations:** 1 Department of Frigid Zone Medicine, College of High Altitude Military Medicine, Army Medical University, Third Military Medical University), Chongqing, China; 2 Key Laboratory of Extreme Environmental Medicine, Ministry of Education of China, Chongqing, China; 3 Key Laboratory of High Altitude Medicine, People’s Liberation Army, Chongqing, China; 4 Department of Emergency Medicine, Shigatse Branch, Xinqiao Hospital, Third Military Medical University, Shigatse, China; 5 Department of Ophthalmology, Southwest Hospital, Army Medical University, Chongqing, China

**Keywords:** Cold-induced vasodilation, Resistance Index for Frostbite, Tibetans, Hans, High altitude

## Abstract

**Background:**

Cold-induced vasodilation (CIVD) reduces the risk of local cold injuries. There are obvious differences in CIVD characteristics among different ethnic groups. This study aimed to compare cold tolerance manifested through CIVD responses in indigenous Tibetans and Han residents in Tibet.

**Methods:**

A total of 12 Tibetans and 10 Han individuals (residence duration at high altitude > 5 years) from high altitudes were recruited in the study. The CIVD test was performed by immersing the participants’ middle finger of the non-dominant hand in cold water (0 ℃) for 30 min followed by recovery for 10 min at a temperature of 25 ± 1 ℃. During the CIVD tests, the participants provided perceptual responses for the immersed hand every 5 min. The participants completed their baseline questionnaires and physiological assessments before the start of the CIVD test, and they completed their hematological tests the following morning.

**Results:**

Compared with Hans at high altitude, Tibetans had higher minimum temperature (*T*_min_), maximum temperature (*T*_max_), and Resistance Index for Frostbite (RIF) scores (p < 0.05), and warmer perceptual responses (*p* < 0.05). We found that red blood cell (RBC) count, hemoglobin (HGB) and hematocrit (HCT) were positively correlated with onset time (Δ*t*_onset_), peak time (Δ*t*_peak_), frequency of CIVD waves (*CIVD*_waves_), and negatively correlated with *T*_min_, *T*_max_, mean finger temperature (*T*_mean_) in the general population (*p* < 0.05), whereas these correlations were not observed in the Tibetan and Han populations (*p* > 0.05). However, the basophil percentage (BAS%), neutrophil percentage (NEU%) and γ-glutamyl transpeptidase (GGT) levels (*p* < 0.05) correlated with the RIF score in Han population.

**Conclusion:**

Compared with Hans at high altitude, indigenous Tibetans demonstrated superior local cold resistance phenotypes in vasomotor regulation, evidenced by their distinct CIVD and perceptual responses. Hematological and biochemical parameters, erythrocytosis is a critical determinant of local cold tolerance at high altitude in the general population. BAS%, NEU%, and GGT are related to local cold tolerance in Han residents.

**Supplementary Information:**

The online version contains supplementary material available at 10.1186/s40101-025-00404-8.

## Background

Individuals residing at high altitudes experience low pressure, hypoxia, cold, dryness, and ultraviolet radiation. Under these extreme conditions, the human body is highly susceptible to the stimulus of cold and hypoxia, which can lead to high altitude sickness and cold injuries. Tibetans have lived in these regions for generations; these have endowed them with exceptional capabilities to adapt to this challenging environment [1]. In contrast, the Han populations need to develop dynamic physiological compensatory mechanisms to adapt to hypoxia and cold exposure at high altitude [2]. Previous studies have focused on the mechanism of acclimatization to hypoxia [3, 4]. Current understanding of the cold tolerance patterns in Han populations residing long-term at high altitudes remains limited.

Cold-induced vasodilation (CIVD) in the fingertips typically occurs 5–10 min after the local cold exposure of the extremities, in which blood vessels in the fingertips vasodilate, and subsequently the peripheral blood flow and temperature of the fingertips increase [5]. This phenomenon may delay the onset, or prevent frostbite, which is beneficial in reducing the risk of local cold injuries and improving manual dexterity under cold exposure. Therefore, strong CIVD responses indicate better local cold resistance capability [6]. Ethnic variations are observed in CIVD responses. The differences in CIVD responses between the Tibetans and high-altitude migrants may reflect their cold tolerance capabilities. However, there are heterogeneous conclusions in existing reports. Tibetans have marginally lower mean skin temperatures during cold water immersion than low-altitude Japanese populations, indicating a blunted CIVD response or stronger vasoconstriction in Tibetans [7]. Conversely, highlanders who demonstrated superior local cold tolerance exhibited higher finger temperatures during cold immersion tests than lowlanders, even when both groups were tested at an altitude of 3500 m [8]. Further studies comparing the characteristics of CIVD between high-altitude native Tibetans and immigrant Han Chinese are needed to reveal their differences for cold tolerance.

Hematological parameters are typically used to evaluate the health status of the body and indicate abnormal metabolism [1]. Changes in routine and biochemical parameters in blood are related to various physiological and pathological processes in the human body. Differences in hematological parameters have been observed between indigenous high-altitude Tibetans and Han individuals who have settled at high altitudes [9]. However, the association between these changes in hematological parameters and CIVD responses remains unknown.

In this study, we investigated the differences in CIVD responses between Tibetan and Han individuals at high altitude, while evaluating a series of physiological, perceptual, and hematological parameters that may contribute to the characteristics of CIVD.

## Methods

### Participants

The study was conducted in December 2024 in Shigatse City (4,200 m), Tibet, China. A total of 12 Tibetans and 10 Han Chinese participants were recruited in this study. Inclusion criteria were as follows: (1) age 20–28 years and male sex; (2) residence duration at high altitude > 5 years; and (3) signed written informed consent. Exclusion criteria were as follows: (1) those with Raynaud’s syndrome; (2) participants who previously experienced local cold injuries; and (3) prescription medication use that would affect vasomotion.

This study is approved by the ethics committee of the Army Medical University with the approval identifier 2022 No 20–02.

### Experimental procedure

The participants were requested to arrive 20 min prior to the start of the procedures. Upon arrival, the participants acclimatized to a room temperature of 25 ± 1 ℃ for 1 h while completing their baseline questionnaires and physiological parameter assessments. During the CIVD tests, the participants provided ratings of hand pain sensation, hand thermal sensation, thermal comfort scales, and affective valence scales of the immersed hand every 5 min [10–13]. The following morning, they completed hematological tests.

### Physiological data collection

Physiological data, including height, weight, heart rate (HR), peripheral capillary oxygen saturation (SpO_2_), regional cerebral oxygen saturation (rSO_2_), skeletal muscle oxygen saturation (SmO_2_), and myoglobin (MYO) levels were obtained. HR and SpO_2_ were measured using a finger-clip pulse oximeter (YX301, Yuwell, China). SmO_2_ and MYO were tested in both the left and right legs simultaneously using a real-time wireless muscle oxygen monitor (Moxy 1-Sensor Bundle, MOXY, America) based on near-infrared spectroscopy. rSO_2_ test was conducted simultaneously on the left and right hemispheres in a quiet and dark environment. The probe of the non-invasive cerebral oximeter (invos 5100 C, SOMANETICS, America) was positioned above the supraorbital ridge on the forehead following an alcohol skin swab.

### CIVD test

The participants were instructed to refrain from smoking, eating, consuming coffee or tea, engaging in physical activity, and cold exposure for 2 h prior to the testing. Alcohol consumption was prohibited for 24 h before the test. Additionally, they were requested to wear the same clothing throughout the testing period.

The CIVD test began with a 5 min stabilization without finger immersion, followed by a 30 min cold water immersion and 10 min recovery period [11]. A thermistor sensor was placed on the lateral side of the distal phalanx of the middle finger of the non-dominant hand and secured with waterproof tape. Subsequently, the forearm was placed on the thigh for 5 min to obtain a baseline measurement. Skin temperature was recorded every second. The participants immersed the middle finger of their non-dominant hand in cold water at 0 ℃ for 30 min (the water covered up to the second knuckle of the finger). After immersion, the finger was removed from the cold water, and the participants placed their left arm on a rolled-up paper towel on the left thigh, with the left wrist suspended in air, and were allowed to continue recovery for 10 min without wiping the finger.

### Measurement of perceptual responses

Based on previous studies, the following perceptual responses were assessed [10–13]: (1) pain scale for the hand ranging from 0 (no pain at all) to 10 (maximal pain conceivable); (2) hand thermal sensation ratings ranging from 0 to 9 (0 (very cold), 4 (neutral), 9 (very hot)); (3) thermal comfort scale ratings ranging from 1 (comfortable) to 5 (extremely uncomfortable); and (4) general affective valence for the hand ranging from − 5 to 5 (− 5 (very bad), 0 (neutral), 5 (very good)).

### Hematological tests

Hematological tests, including routine blood tests and biochemical analyses, were conducted using fasting blood samples collected by trained nurses. The following parameters were assessed for each participant at the 953rd Hospital of People’s Liberation Army: red blood cell (RBC) count, hemoglobin (HGB), mean corpuscular hemoglobin, mean corpuscular HGB concentration, white blood cell count, monocyte count, hematocrit (HCT), coefficient of variation of red cell distribution width (RDW), standard deviation of RDW, lymphocyte count, mean corpuscular volume, mean platelet volume, basophil count (BAS), eosinophil count, neutrophil count (NEU), platelet distribution width, platelet count, plateletcrit, platelet-to-larger cell ratio, γ-glutamyl transpeptidase (GGT), alanine aminotransferase, alkaline phosphatase, aspartate aminotransferase, total bile acid, total bilirubin, direct bilirubin, indirect bilirubin, total protein, creatinine, urea, and uric acid.

### Measurements and calculations

**Fig. 1:**
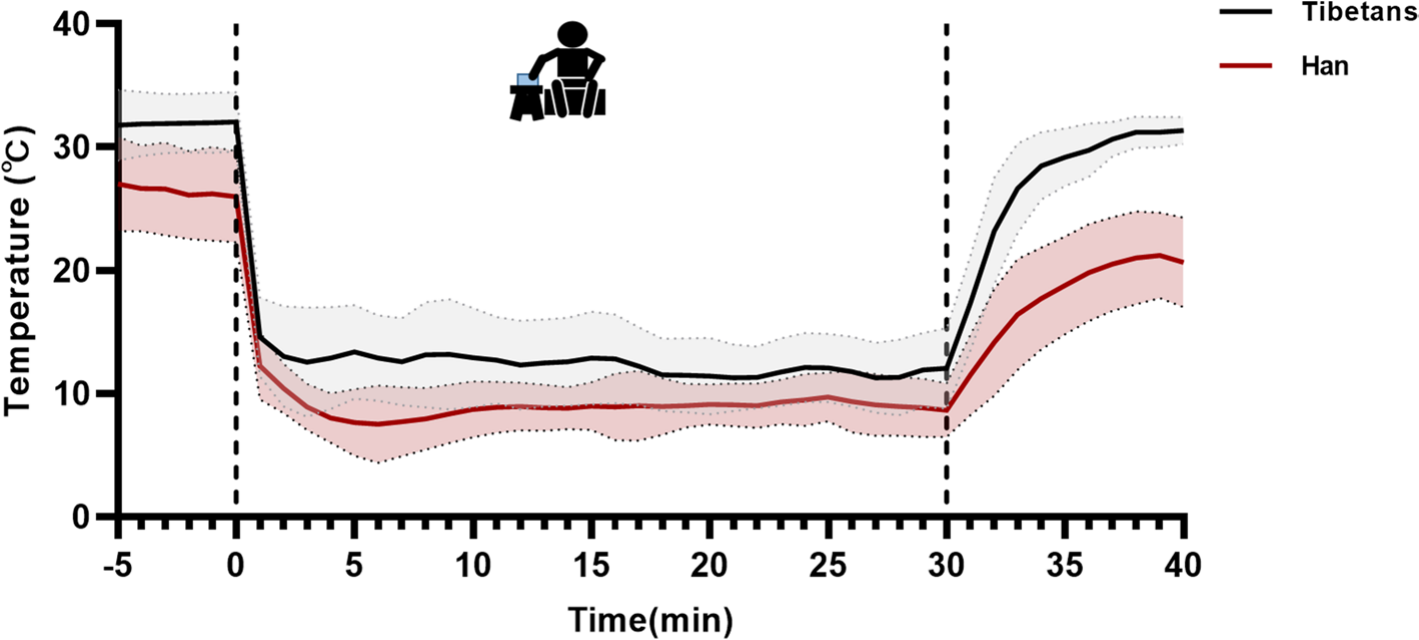
Mean (± SD) finger temperature during rest, immersion, and recovery between Tibetans and Hans

Individual CIVD response curves for each participant were generated using the raw thermistor temperature data of the finger and plotted as a function of time (min) (Fig. 1). Parameters that defined the strength of the CIVD response were calculated from the middle finger data using the methods described by Daanen (2003) and included the following [5]: (1) minimum temperature (*T*_min_) is the lowest recorded skin temperature of the finger before CIVD onset; (2) maximum temperature (*T*_max_) is the highest recorded skin temperature of the finger during CIVD; (3) onset time (Δ*t*_onset_) is the time from immersion to *T*_min_; (4) amplitude (*T*_*ampl*_) is the difference between *T*_min_ and *T*_max_; (5) peak time (Δ*t*_peak_) is the time interval between *T*_min_ and *T*_max_; (6) mean skin temperature of the finger (*T*_mean_) denotes the skin temperature of the finger averaged over the 5–30 min immersion period; (7) frequency of CIVD waves (*CIVD*_waves_) during the 30 min immersion is defined by a ≥ 1 ℃ increase in temperature, followed by a visible, complete wave pattern including both an increasing and decreasing; (8) rewarming speed (Rewa speed) is the rate of temperature increase during the recovery period.

The Resistance Index for Frostbite (RIF), an indicator for quantifying the intensity of the CIVD response, is determined by adding the points of the *T*_min,_ *T*_max_ and Δ*t*_onset_ together for each individual [14]. The RIF ranges between 3 and 9, wherein 3 is the lowest score and indicates a weak reaction to cold temperature, and 9 is the highest score indicating a strong reaction to cold temperature.

### Statistical analysis

Statistical analysis was performed using SPSS 21.0 Statistics. Data are presented as mean ± standard deviation, and statistical significance was set at *p* < 0.05. The Student’s *t*-test analysis was used to compare quantitative variables between the Tibetans and Han individuals. To investigate the relationship of CIVD parameters and hematologic parameters, the Spearman and Pearson correlation analyses were performed. The Spearman’s correlation analysis was used for non-parametric variables, and the Pearson’s correlation analysis was used for continuous variables.

## Results

### Baseline characteristic of participants

Baseline characteristic of the participants is shown in Table 1. Mean age among the Tibetan and Han populations was 24.00 ± 2.45 and 26.50 ± 1.35 years, respectively, and mean body mass indices of the two groups were 24.57 ± 8.10 and 22.34 ± 2.26 kg/m^2^. The physiological parameters, HR, SpO_2_, left-rSO_2_, right-rSO_2_, left-SmO_2_, right-SmO_2_, left-MYO, and right-MYO had no significant difference between the two groups (*p* > 0.05).

**Table 1:** Baseline characteristics of the study population

	Tibetans (*n* = 12)	Hans (*n* = 10)	*p*-value
Age (years)	24.00 ± 2.45	26.50 ± 1.35	0.259
Height (cm)	174.58 ± 8.25	173.20 ± 6.09	0.441
Weight (kg)	75.01 ± 26.46	66.90 ± 6.31	0.097
BMI (kg/m^2^)	24.57 ± 8.10	22.34 ± 2.26	0.109
Current smoker (*n*, %)	9 (75.00%)	4 (40.00%)	0.192
HR (beats/min)	80.83 ± 12.63	78.60 ± 12.79	0.695
SpO_2_ (%)	86.08 ± 2.39	86.00 ± 2.49	0.884
L-rSO_2_ (%)	62.33 ± 4.85	70.90 ± 6.62	0.202
R-rSO_2_ (%)	61.75 ± 5.21	70.00 ± 7.26	0.433
L-SmO_2_ (%)	49.25 ± 11.83	58.90 ± 9.70	0.231
R-SmO_2_ (%)	49.92 ± 11.99	57.90 ± 11.78	0.712
L-MYO (g/dL)	12.53 ± 0.32	12.35 ± 0.34	0.983
R-MYO (g/dL)	12.46 ± 0.39	12.33 ± 0.36	0.263

*HR* heart rate at resting, *SpO*_*2*_ peripheral capillary oxygen saturation, *L-rSO*_*2*_ left regional cerebral oxygen saturation, *R-rSO*_*2*_ right regional cerebral oxygen saturation, *L-SmO*_*2*_ left-leg skeletal muscle oxygen saturation, *R-SmO*_*2*_ right-leg skeletal muscle oxygen saturation, *L-MYO* left-leg myoglobin, *R-MYO* right-leg myoglobin

### CIVD parameters on the finger

CIVD response of finger skin temperature in Tibetans and Han is shown in Fig. 1. The finger temperature in Tibetans was higher than in Han individuals during rest, immersion, and recovery. Results of the CIVD parameters are presented in Table 2; *T*_min_, *T*_max_, and the RIF score had significant differences between the Tibetans and Han Chinese (*p* < 0.05). *T*_min_ was about 4.49 ℃ higher among the Tibetans (11.32 ± 3.73 ℃) than that among the Han population (6.83 ± 2.47 ℃). Similarly, *T*_max_ was about 4.31℃ higher among the Tibetans (14.38 ± 3.75 ℃) than that among the Han population (10.07 ± 2.21 ℃). The Tibetans (8.75 ± 0.62) had higher RIF scores than the Han population (8.20 ± 0.92). However, other CIVD parameters, including Δ*t*_onset_, *T*_mean_, *T*_*ampl*_, *CIVD*_waves_, Rewa speed, and Δ*t*_peak_ showed no significant differences between the Tibetans and Han Chinese.

**Table 2:** Cold-induced vasodilation parameters in Tibetans and Han

	Tibetans (*n* = 12)	Hans (*n* = 10)	*p*-value
CIVD parameters			
*T*_min_ (℃)	11.32 ± 3.73	6.83 ± 2.47	0.008*
*T*_max_ (℃)	14.38 ± 3.75	10.07 ± 2.21	0.011*
Δ*t*_onset_(min)	4.58 ± 3.37	8.00 ± 4.83	0.244
*T*_mean_ (℃)	12.16 ± 2.63	8.85 ± 1.97	0.193
*T*_ampl_ (℃)	3.08 ± 1.75	3.29 ± 1.40	0.593
*CIVD*_wave_ (#)	3.08 ± 0.67	1.80 ± 1.03	0.147
Rewa speed (°C/min)	1.40 ± 0.37	0.91 ± 0.28	0.345
Δ*t*_peak_ (min)	3.08 ± 1.56	6.00 ± 2.54	0.109
RIF score	8.75 ± 0.62	8.20 ± 0.92	0.041*

^*^Indicate significant statistical differences from Hans

### Perceptual responses

Perceived pain, thermal sensation, thermal comfort, and affective valence were significantly different between the Tibetans and Han individuals (Table 3). The score for perceived pain in the immersed hand at 15 min, 20 min, and recovery for 5 min showed a significant difference between the two groups (*p* < 0.05). The Tibetans had a lower score for perceived pain than the Han population. This observation suggests that the Tibetans had a relatively lower sensitivity to pain. Additionally, the Tibetans had higher thermal sensation scores than the Han population in the immersed hand at 15 and 30 min (*p* < 0.05), which indicates that the Tibetans had a relatively higher thermal sensation. The Tibetans had a lower thermal comfort score than the Han population in the immersed hand at 30 min, 5 min recovery, and 10 min (*p* < 0.05). This result suggests that the Tibetans had better thermal comfort. In particular, the Tibetans had higher affective valence than the Han individuals during the rest, immersed hand at 10 min, 5 min recovery, and 10 min (*p* < 0.05), indicating that the Tibetans had better affective valence than the Han population.

**Table 3:** Tibetans and Han perceptual measurements in cold-induced vasodilation test

		Rest	0 ℃ immersion						Recovery	
		0 min	5 min	10 min	15 min	20 min	25 min	30 min	5 min	10 min
Score of perceived pain	Tibetan	0.00 ± 0.00	1.25 ± 2.53	0.75 ± 0.87	0.92 ± 1.24*	1.00 ± 1.71*	0.83 ± 1.59	0.92 ± 1.78	0.00 ± 0.00*	0.00 ± 0.00
	Hans	0.00 ± 0.00	2.80 ± 1.81	2.00 ± 1.49	1.30 ± 0.67	1.40 ± 0.70	1.50 ± 0.71	1.70 ± 0.95	0.80 ± 0.79	0.00 ± 0.00
Score of thermal sensation scale	Tibetan	5.50 ± 1.09	4.25 ± 1.06	4.25 ± 1.06	4.33 ± 1.37*	4.50 ± 0.80	4.75 ± 1.06	4.33 ± 1.15*	5.83 ± 1.11	5.92 ± 1.08
	Hans	4.00 ± 1.15	2.60 ± 1.51	2.40 ± 0.70	2.30 ± 0.67	2.20 ± 1.03	2.70 ± 1.42	1.90 ± 0.57	3.90 ± 0.88	4.30 ± 0.82
Score of thermal comfort scale	Tibetan	1.00 ± 0.00	1.25 ± 0.45	1.33 ± 0.49	1.25 ± 0.45	1.17 ± 0.58	1.08 ± 0.29	1.17 ± 0.39*	1.00 ± 0.00*	1.00 ± 0.00*
	Hans	1.00 ± 0.00	2.80 ± 0.79	2.20 ± 0.42	2.30 ± 0.67	2.20 ± 0.63	2.00 ± 0.47	2.30 ± 0.67	1.40 ± 0.52	1.10 ± 0.32
Score of affective valence scale	Tibetan	5.00 ± 0.00*	4.75 ± 0.45	4.83 ± 0.39*	4.75 ± 0.62	4.67 ± 0.65	4.58 ± 0.67	4.42 ± 0.67	5.00 ± 0.00*	5.00 ± 0.00*
	Hans	3.30 ± 1.60	2.70 ± 0.95	3.00 ± 1.41	2.00 ± 1.05	2.40 ± 0.97	2.50 ± 0.71	2.60 ± 1.26	2.80 ± 1.03	2.70 ± 0.95

^*^Indicate significant statistical differences from Hans

### Hematological parameters and correlation analysis

**Fig. 2:**
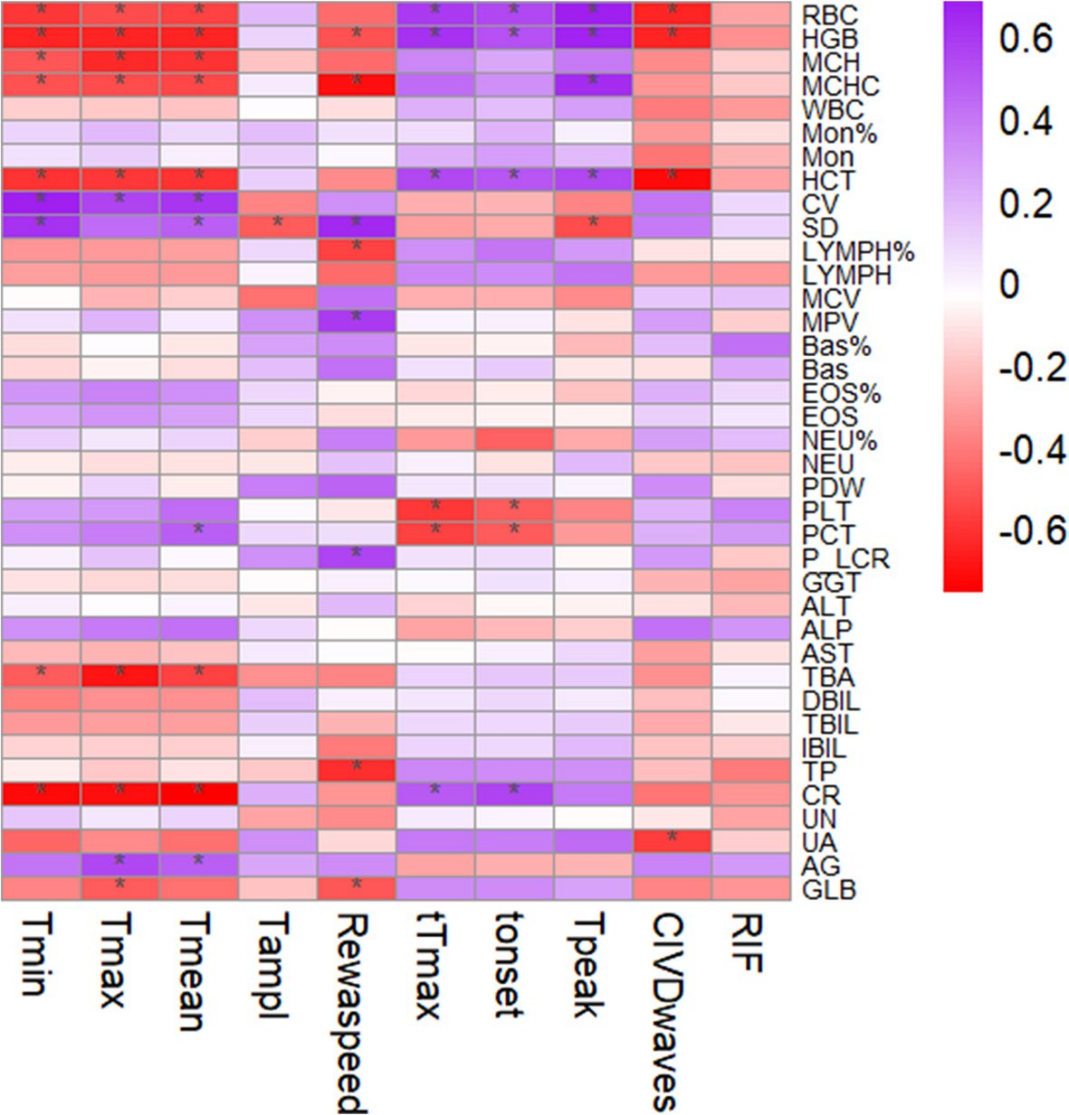
Relationship between hematologic parameters and cold-induced vasodilation parameters in the general population. *Indicate significant statistical differences

No statistically significant difference was observed in the hematological parameters between the Tibetans and Han population (Table 4). The relationship between CIVD and hematological parameters among the general population, Tibetans, and Han population is shown in Fig. 2, Additional files 1 and 2. Additionally, we observed that RBC, HGB, and HCT were positively correlated with Δ*t*_onset_, Δ*t*_peak_, *CIVD*_waves_, and negatively correlated with *T*_min_, *T*_max_, *T*_mean_ in the general population. The correlation between the RIF score and hematological parameters was observed only in the Han population, but not among the Tibetans and the general population. In the Han population, the primary results showed a positive correlation between the RIF score and BAS% (*p* < 0.05), and a negative correlation between the RIF score and NEU% (*p* < 0.05) and γ-glutamyl transpeptidase (GGT) (*p* < 0.05) (Additional files. 2).

**Table 4:** Hematologic parameters results in Tibetans and Hans

	Tibetans (*n* = 12)	Hans (*n* = 10)	*p*-value
RBC (10^12^/L)	5.26 ± 0.52	6.35 ± 0.51	0.423
HGB (g/L)	162.80 ± 20.92	206.62 ± 13.72	0.107
MCH (pg)	30.88 ± 1.59	32.56 ± 1.24	0.548
MCHC (g/L)	344.60 ± 14.28	366.63 ± 12.14	0.470
WBC (10^9^/L)	6.58 ± 1.33	7.16 ± 1.31	0.788
MON (%)	5.84 ± 3.10	7.21 ± 3.99	0.708
MON (10^9^/L)	0.38 ± 0.22	0.52 ± 0.28	0.618
HCT (%)	47.18 ± 5.22	56.35 ± 3.88	0.089
RDW-CV (%)	13.40 ± 0.84	12.62 ± 0.744	0.774
RDW-SD (%)	43.60 ± 2.72	40.88 ± 2.10	0.261
LYMPH (%)	36.64 ± 4.74	43.19 ± 3.53	0.191
LYMPH (10^9^/L)	2.41 ± 0.57	3.09 ± 0.57	0.934
MCV (fL)	89.59 ± 2.58	88.73 ± 2.13	0.629
MPV (fL)	10.86 ± 0.38	10.61 ± 1.00	0.139
BAS (%)	0.24 ± 0.16	0.21 ± 0.11	0.567
BAS (10^9^/L)	0.01 ± 0.01	0.01 ± 0.00	0.227
EOS (%)	2.27 ± 1.59	2.06 ± 2.34	0.326
EOS (10^9^/L)	0.15 ± 0.12	0.16 ± 0.19	0.180
NEU (%)	55.17 ± 6.76	47.43 ± 6.62	0.841
NEU (10^9^/L)	3.63 ± 0.94	3.39 ± 0.73	0.743
PDW (%)	12.69 ± 0.89	12.76 ± 2.20	0.077
PLT (10^9^/L)	271.70 ± 50.85	239.00 ± 40.01	0.873
PCT (%)	0.29 ± 0.05	0.25 ± 0.04	0.476
P_LCR (%)	31.42 ± 3.12	29.91 ± 8.47	0.108
GGT (IU/L)	33.09 ± 21.95	34.63 ± 23.07	0.821
ALT (IU/L)	32.64 ± 13.24	30.88 ± 26.25	0.112
ALP (IU/L)	93.27 ± 20.06	81.00 ± 23.70	0.584
AST (IU/L)	26.91 ± 7.12	30.13 ± 10.44	0.314
TBA (μmol/L)	2.31 ± 1.94	4.04 ± 3.02	0.233
DBIL (μmol/L)	6.35 ± 3.65	9.34 ± 3.42	0.996
TBIL (μmol/L)	18.34 ± 6.02	25.43 ± 5.15	0.533
IBIL (μmol/L)	11.92 ± 3.33	16.09 ± 4.13	0.368
TP (g/L)	75.21 ± 3.87	80.83 ± 3.75	0.998
CR (μmol/L)	72.64 ± 10.18	87.88 ± 9.79	0.973
UN (mmol/L)	6.15 ± 1.23	6.49 ± 1.90	0.305
UA (mmol/L)	401.18 ± 68.29	509.38 ± 121.84	0.147

*RBC* red blood cell count, *HGB* hemoglobin, *MCH* mean corpuscular hemoglobin, *MCHC* mean corpuscular HGB concentration, *WBC* white blood cell count, *MON* monocyte count, *HCT* hematocrit, *RDW-CV* coefficient of variation of RDW, *RDW-SD* standard deviation of RDW, *LYMPH* lymphocyte count, *MCV* mean corpuscular volume, *MPV* mean platelet volume, *BAS* basophil count, *EOS* eosinophil count, *NEU* neutrophil count, *PDW* platelet distribution width, *PLT* platelet count, *PCT* plateletcrit, *P_LCR* platelet-to-larger cell ratio, *GGT* γ-glutamyl transpeptidase, *ALT* alanine aminotransferase, *ALP* alkaline phosphatase, *AST* aspartate aminotransferase, *TBA* total bile acid, *TBIL* total bilirubin, *DBIL* direct bilirubin, *IBIL* indirect bilirubin, *TP* total protein, *CR* creatinine, *UN* urea, *UA* uric acid

## Discussion

Differences in adaptability to high altitude environments among the Han and Tibetan individuals are owing to their hypoxia responses and physical phenotypes. The characteristics of their responses in cold conditions have not been investigated. Therefore, the CIVD response is used as an index for local cold tolerance, and different ethnic populations exhibit distinct characteristics in this response [6]. In this study, we demonstrated the differences in CIVD parameters and perceptual response between the Tibetans and Han Chinese. Our results suggest that the Tibetans had greater cold tolerance than that of Hans, with differences in CIVD responses. Additionally, we observed that RBC, HGB, and HCT were positively correlated with Δ*t*_onset_, Δ*t*_peak_, *CIVD*_waves_, and negatively correlated with *T*_min_, *T*_max_, and *T*_mean_ in the general population, indicating that alterations in erythrocyte indices may serve as the principal determinants of local cold tolerance at high altitude. Finally, the RIF scores correlated with BAS%, NEU%, and GGT in the Han population more than those among the Tibetans, which suggests potential mechanisms for understanding the local cold tolerance capacities of high-altitude residents.

CIVD responses and RIF scores can serve as valuable predictive tools for assessing an individual’s susceptibility to frostbite [16]. Tibetans are considered the best adapted ethnic group to high-altitude environments. This observation is reflected by their better oxygen metabolism and cold tolerance. Most high-altitude residents exhibit higher BMRs [16]. Specifically, studies have reported that the Tibetans have higher BMRs than those estimated using the World Health Organization references [16]. The higher BMRs suggest a better oxygen metabolism and thermogenic capacity among Tibetans, which are helpful in reducing the risk of local cold injury. Peripheral skin temperatures of high-altitude Tibetans in Nepal were reported to be higher than those of the Andean population in Bolivia, despite similar living conditions at high altitudes and excellent blood flow and cold tolerance exhibited by both groups [16]. However, a comparative study revealed that Tibetans had slightly lower mean skin temperatures during finger cold water immersion than that of low-altitude Japanese populations [7]. These contentious results imply that relying solely on mean skin temperatures is inadequate to characterize the physiological profile of CIVD and evaluate cold tolerance in high altitudes.

In our study, several parameters defining the strength of the CIVD response were analyzed. We found that the *T*_min_, *T*_max_, and RIF scores were better for the Han population at high altitude. *T*_min_ and *T*_max_ define skin temperature during cold-water immersion and directly correlate with pain perception [10]. Studies indicate that pain occurs when fingertip temperature drops below 10 °C, and numbness develops at − 5° C [16, 16]. Pain sensation both serves as a warning for frostbite and a reflection of heightened cold sensitivity [10]. Our data revealed that the Tibetans rest finger temperatures were higher than those of Hans; consistently maintained above 11 °C during immersion, with peaks exceeding 14 °C, whereas finger temperatures of Han migrants ranged between 6 and 10 °C. Correspondingly, the Tibetans demonstrated more favorable perceptual responses during the CIVD testing than the Han participants. Typically, individuals who have acclimatized to high-altitude environments tend to experience less cold sensation, pain, and discomfort in cold conditions than those who have not acclimatized to these conditions. Therefore, our results indicated that Tibetans have a stronger CIVD response than Han migrants, confirming that the superior local cold tolerance among Tibetans is a result of evolutionary adaptation. Han migrants can gradually adapt to the plateau over the years; they do not achieve the same level of cold tolerance as Tibetans. Thus, elucidating the regulatory factors and mechanisms of CIVD is vital for improving high-altitude resilience in Han populations.

Erythrocytosis is a key manifestation of high-altitude acclimatization, reflected by persistently elevated RBC count, HGB concentration, and HCT that serve a compensatory role in sustaining tissue oxygenation [9]. In the general population, we observed that RBC, HGB, and HCT were positively correlated with Δ*t*_onset_, Δ*t*_peak_, *CIVD*_waves_, and negatively correlated with *T*_min_, *T*_max_, *T*_mean_, suggesting that erythrocytosis is a critical determinant of local cold tolerance at high altitude. We speculated that increased blood viscosity may indirectly compromise peripheral circulation in the extremities, thereby diminishing local cold tolerance. In our study, we observed a trend of higher HGB and HCT levels in the Han individuals than in the Tibetans, which is consistent with the findings of other studies [3, 9, 16]. This phenomenon presumably reflects an adaptive strategy to high-altitude hypoxia through augmented oxygen-carrying capacity; however, it is accompanied by a pronounced increase in blood viscosity. Differences in erythroid indices and their governing regulatory mechanisms are likely to underpin the divergent CIVD responses observed between Tibetans and Hans under cold stress. In addition, Tibetans living at high altitudes have greater blood flow owing to higher NO levels than those living at low altitudes [16], which increases the transit time of RBC to tissues, enhances the diffusion of oxygen content, and indirectly increases blood circulation to the limbs, potentially improving tolerance to peripheral cold and increasing skin temperature [16]. However, we did not observe significant associations between erythroid indices and CIVD parameters in Tibetans and Hans. Therefore, analysis of the factors influencing CIVD among other hematological parameters may help identify the limiting factors of cold acclimatization among Han immigrants at high altitudes.

Our study revealed that RIF scores were associated with BAS%, NEU%, and GGT among Han participants. The Han population exhibited a downward trend in both BAS% and NEU% when compared with Tibetans, which is consistent with previous findings [9]. The results were likely attributable to hypobaric hypoxia. Hypoxia not only directly impairs immune-cell proliferation, differentiation, and induces apoptosis but also compromises cellular energy supply and weakens immunity [9]. The reduction in BAS% and NEU% indicates heightened immune stress among Han individuals at high altitude and may represent a reason underlying their diminished local cold tolerance. BAS contain abundant histamine, which is released when they are activated. Previous studies on healthy volunteers have supported the role of histamine-induced dose-dependent veno-dilatation in the hands, vasodilation in the skin microcirculation, and systemic responses [16]. We conclude that BAS may indirectly induce vasodilation, which could be related to the release of vasodilatory substances in the potential mechanisms of CIVD. Vasoconstriction typically reduces heat loss from the extremities, but tissue perfusion may be compromised if the rheological properties of the blood are impaired. NEU modified their mechanical and/or adhesive properties in the cold, and this may occlude microvessels, thereby leading to thromboembolism, frostbite, and other diseases prevalent in winter [16]. This study showed that NEU may affect blood fluidity in the cold environment, which could indirectly affect the CIVD response. In addition, GGT was also related to RIF. In a previous study, GGT was an early and sensitive biomarker of oxidative stress [16], which indicated that the excessive levels of GGT might potentially have adverse effects on the body at high altitude. Recent findings have demonstrated a strong inverse relationship between serum GGT and endothelium-dependent vasodilation, which is consistent with the results of our study [16].

Our study provides new insights into the adaptive differences between Tibetans and Han individuals for cold tolerance at high altitudes. Moreover, it accounts for the role of hematological parameters, offering suggestions for future research areas. The limitations of our study include a small sample size and the focus on young men only, which restricts the generalizability of the study to a broader population. Additionally, our analysis is limited to correlational studies of blood samples. In our next study, we will expand the sample size and conduct omics analysis on blood samples to further investigate the underlying mechanisms.

## Conclusions

Tibetans and Han Chinese at high altitude exhibit differences in CIVD parameters and their perceptual responses to cold stimuli. Specifically, Tibetans demonstrate superior cold tolerance compared to Han individuals at high altitude. Furthermore, hematological and biochemical parameters, erythrocytosis, are critical determinants of local cold tolerance at high altitude in the general population. BAS%, NEU%, and GGT are related to local cold tolerance in Han residents. Therefore, this study lays the foundation for further exploration of cold tolerance among residents of high-altitude cold regions.

## Supplementary Information

Additional file 1. Relationship between Hematologic parameters and Cold-induced vasodilation parameters in Tibetans. *indicate significant statistical differences

Additional file 2. Relationship between Hematologic parameters and Cold-induced vasodilation parameters in Hans. *indicate significant statistical differences

## Data Availability

No datasets were generated or analysed during the current study.

## Abbreviations

CIVD: Cold-induced vasodilation
*T*_min_: Minimum temperature
*T*_max_: Maximum temperature
RIF: Resistance Index for Frostbite score
BAS%: Basophil percentage
NEU%: Neutrophil percentage
GGT: γ-Glutamyl transpeptidase
HR: Heart rate
SpO_2_: Peripheral capillary oxygen saturation
rSO_2_: Regional cerebral oxygen saturation
SmO_2_: Skeletal muscle oxygen saturation
MYO: Myoglobin
RBC: Red blood cell
HGB: Hemoglobin
HCT: Hematocrit
RDW: Red cell distribution width
Δ*t*_onset_: Onset time
*T*_*ampl*_: Amplitude
Δ*t*_peak_: Peak time
*T*_mean_: Mean finger skin temperature
Rewa speed: Rewarming speed
BMRs: Basal metabolic rates
