# Supplementary figures and images for: Characteristics of cold-induced vasodilation among Tibetans and Han Chinese at high altitudes
Source: J Physiol Anthropol. 2025 Sep 30;44:24. doi: 10.1186/s40101-025-00404-8 (PMC12487105; doi:10.1186/s40101-025-00404-8)

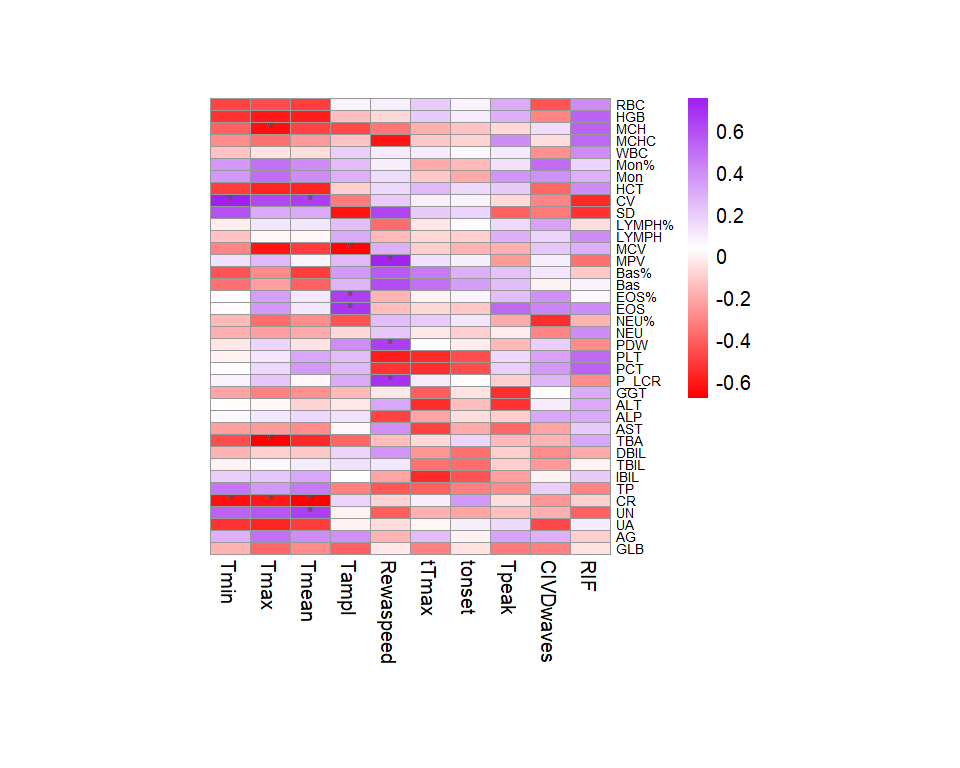

Supplement: Supplementary file 1 — Additional file 1. Relationship between Hematologic parameters and Cold-induced vasodilation parameters in Tibetans. *indicate significant statistical differences [file 40101_2025_404_MOESM1_ESM.tiff]

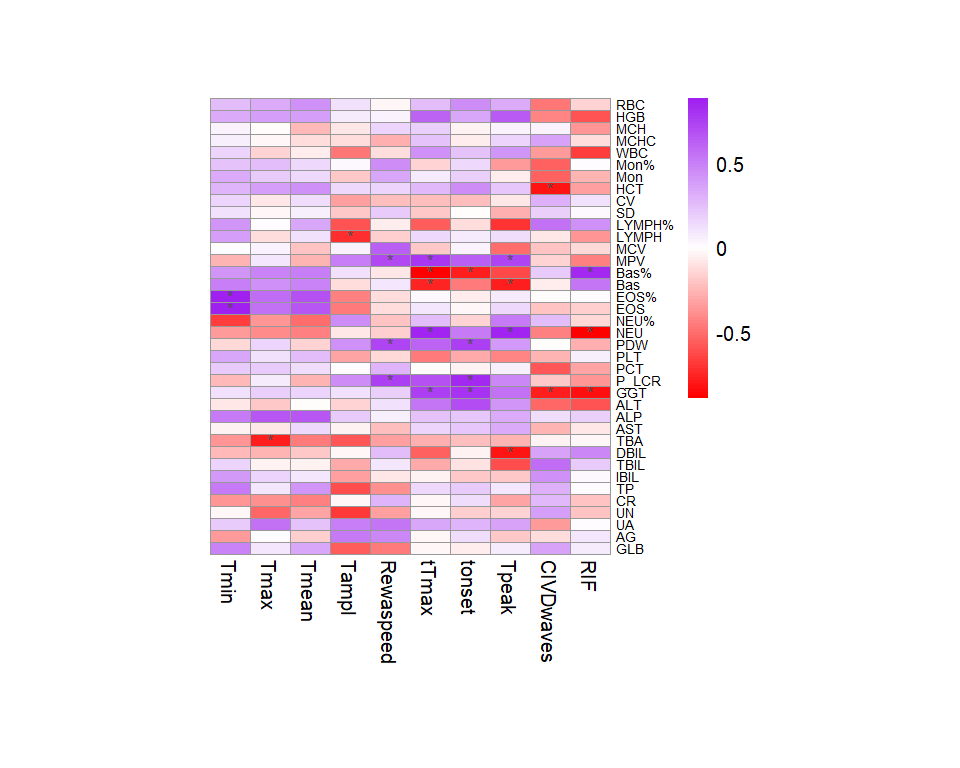

Supplement: Supplementary file 2 — Additional file 2. Relationship between Hematologic parameters and Cold-induced vasodilation parameters in Hans. *indicate significant statistical differences [file 40101_2025_404_MOESM2_ESM.tiff]
